# Aerial application of copper for dothistroma control in New Zealand’s planted forests—effect on stream environments

**DOI:** 10.1007/s11356-017-0020-4

**Published:** 2017-09-13

**Authors:** Brenda R. Baillie, Anthony W. Evanson, Diana Unsworth, Sunita Jeram

**Affiliations:** 1 Scion, Private Bag 3020, Rotorua, 3046 New Zealand; 2 NSW Department of Primary Industries, 1243 Bruxner Highway, Wollongbar, NSW 2477 Australia

**Keywords:** Copper, Water quality, Sediment, Dothistroma, Planted forest, New Zealand, Aerial application

## Abstract

Limited information is available on the risk to aquatic environments from the aerial application of copper fungicides to treat dothistroma needle blight in managed forests. Cuprous oxide was aerially applied to three catchments of *Pinus radiata* of varying age classes in the central North Island of New Zealand. Copper was monitored in stream water and sediments prior to and for 1 month after application. Copper deposits collected from tracer plates deployed above the water surface along the stream channels within the treated areas at each site ranged from 13 to 406 ppm. Lowest concentrations occurred above small stream channels with dense overhead riparian vegetation. Peak copper concentrations in stream water across the three sites ranged from 28 to 60 μg L^−1^ and were below the analytical detection limit within hours. Copper concentrations were higher and persisted for longer in stream sediment (range 1.7–6.1 mg kg^−1^, sampled at two sites only). Copper concentrations in sediments were below environmental guidelines. Copper concentrations in water and sediment indicated a low risk to aquatic organisms based on the exposure times to the concentrations measured in this study.

## Introduction

Copper is a naturally occurring metallic element found in the earth’s crust and is an essential trace element required in very small quantities to support the functioning of biological systems of all living organisms. However, both deficient and excess concentrations of copper in the environment can be harmful (Environmental Protection Agency 2007; Soetan et al. 2010; Kiaune and Singhasemanon 2011). Humans have been using copper for more than 10,000 years, and today it is mined and used extensively around the world in the electrical, electronics, communication, industrial construction, and transportation industries (International Copper Study Group 1997). Copper has also been used as a fungicide from as far back as the eighteenth century to control a wide range of diseases found in horticultural and agricultural crops (Morton and Staub 2008).

Copper fungicides are also used in the forest industry to control dothistroma needle blight (dothistroma) in pines (*Pinus* spp.), particularly *Dothistroma septosporum* (Bulman et al. 2016). Dothistroma causes premature defoliation in pines, although the natural susceptibility to the disease varies among pine species. In more serious outbreaks, the disease has a direct impact on tree growth, and in severely infected stands, tree mortality can occur. Dothistroma was first identified as a serious issue for planted forests in North America in the 1950s. In recent decades, a number of reports have highlighted the increasing prevalence of the disease and an increase in the severity of outbreaks (Barnes et al. 2008; Welsh et al. 2009; Barnes et al. 2014), particularly in the northern hemisphere forests which authors suggest may be attributable to underlying changes in weather patterns (Welsh et al. 2014; Woods et al. 2016).

In many regions, where disease levels of dothistroma are low, management interventions to control the disease are not economically justifiable. For more serious disease outbreaks, along with the use of copper fungicides, interventions such as timing of silvicultural treatments, and breeding and planting of disease resistance species are often used in combination to control dothistroma. In the northern hemisphere, the use of copper fungicides to control dothistroma is mainly confined to forest nurseries (Bulman et al. 2016). This is due, in part, to increasing pressure to reduce or eliminate overall pesticide use (including fungicides) in forests, driven by factors such as increasing public concerns regarding the social and environmental risks, forest certification requirements, and governmental regulations restricting or prohibiting pesticide use (Forest Stewardship Council 2005; Forestry Commission 2011). Treatment of the disease with aerial application of copper fungicides has been particularly important in New Zealand and Australian planted forests, where infection levels can impact on the productivity and economic viability of the timber crop (Bradshaw 2004; Bulman et al. 2008, 2016).

In New Zealand, copper is aerially applied to control dothistroma in *Pinus radiata* stands, the main timber species grown there (Bulman et al. 2008; Forest Owners Association & Ministry for Primary Industries unknown). The area of forest sprayed varies markedly from year to year (from less than 10,000 ha to over 180,000 ha) depending on infection levels (Bulman et al. 2004). This disease is considered to be one of the more serious affecting New Zealand’s planted forests and estimated costs to the industry in reduced productivity and disease control have been estimated at just under $20 million per year (Watt et al. 2011). While silvicultural treatments such as thinning and pruning reduce favorable conditions for incubation and spread of the disease, aerial treatment with copper fungicide remains the most effective method of control (Bulman et al. 2004, 2016).

Although copper commonly occurs as a natural metal in surface water bodies, anthropogenic activities that introduce excess quantities of copper into receiving freshwater environments can pose a risk to aquatic organisms (Hickey and Clements 1998; Jarvinen and Ankley 1999; Kiaune and Singhasemanon 2011; European Food Safety Authority 2013). The process of copper speciation and adsorption in surface waters and sediments is complex. Most of the dissolved copper in water (copper that has not adsorbed to particulate matter) binds to organic ligands and sediment provides an important sink and storage site for copper, reducing the amount of bioavailable copper in the system (Kiaune and Singhasemanon 2011). It is the bioavailable component of dissolved copper (free Cu^2+^ and Cu^+^ ions) that is potentially toxic to aquatic organisms, if present in sufficiently high levels (Kiaune and Singhasemanon 2011). Water quality characteristics such as the amount of dissolved organic carbon, pH, and water hardness can influence the amount of biologically available copper in the aquatic environment.

The mechanisms for uptake and processing copper and the level of sensitivity to copper toxicity vary among aquatic organisms (Jarvinen and Ankley 1999; Environmental Protection Agency 2007; Kiaune and Singhasemanon 2011; MacBean 2012). Copper is readily accumulated by aquatic organisms, but as copper is an essential element, it is usually regulated and only becomes toxic when accumulation rates exceed detoxification and excretion rates (ANZECC 2000; Kiaune and Singhasemanon 2011). Some algal species and aquatic invertebrates such as mollusks tend to show the least sensitivity to copper, whereas more sensitive species such as *Daphnia* and rainbow trout (*Oncorhynchus mykiss*) are typically used to assess the toxicity of copper in the aquatic environment (Jarvinen and Ankley 1999; Kiaune and Singhasemanon 2011; MacBean 2012; European Food Safety Authority 2013). A range of sub-lethal effects have been observed in aquatic organisms, including reductions in benthic invertebrate diversity and abundance, synergistic effects of copper and iron on the condition of the common eel (*Anguilla anguilla*), and the impairment of fish chemosensory mechanisms (Canadian Council of Ministers of the Environment 1999; Kiaune and Singhasemanon 2011; Esteve et al. 2012).

Because of the potential risk of excess copper to the aquatic environment, environmental guidelines have been established in some countries to safeguard aquatic ecosystems (i.e., ANZECC 2000; Canadian Council of Ministers of the Environment 2007; Environmental Protection Agency 2007). In addition, the Forest Stewardship Council (FSC), a global organization that provides a forest certification scheme aimed at promoting well-managed forests, has recently placed cuprous oxide (CAS No. 1317-39-1) on the FSC highly hazardous pesticide list because of its aquatic toxicity (Forest Stewardship Council 2015a, 2015b). FSC currently certifies 190,695,831 ha of forests worldwide and is the main certifier of planted forests in New Zealand (1.24 million ha, 70% of the planted forest estate) (Forest Owners Association & Ministry for Primary Industries unknown; Forest Stewardship Council 2016).

In a New Zealand study on the aquatic fate of copper aerially applied to control dothistroma in a central North Island forest, where samples were taken approximately 1 week later, copper concentrations were below the analytical detection limits in stream water (50 μg L^−1^), but higher concentrations were detected in the fine organic matter, whereas no identifiable changes in copper concentration were observed in aquatic invertebrates (Fish 1968). Data from two unpublished studies, one in a central North Island forest (1976) and the other in a West Coast, South Island forest (1980), measured peak concentrations in stream water of up to 300 μg L^−1^ on the day of aerial copper application, which declined to either just above or below analytical detection limits (not defined) within 48 h after spraying (Collier and Hickey 1998; unpublished data, P. Beets, Scion, Rotorua, New Zealand). A desktop review of the potential effects of copper on aquatic organisms in New Zealand indicated that under some scenarios, particularly direct spray onto waterways, there was the potential for copper to reach concentrations for a duration likely to affect the more sensitive aquatic species such as the mayfly (*Deleatidium* spp.) and *Daphnia* (Collier and Hickey 1998; Hickey and Clements 1998).

Both the published and unpublished information available on the fate of copper in the waterways of New Zealand’s planted forests are sparse and dated. Quantifying the effects of aerial copper application on water quality under current forest management practices would assist the forest industry, international and governmental administrative bodies, and regulatory agencies to make informed decisions on the risks and benefits of using aerial application of copper to control dothistroma.

The objective of this study was to measure the concentrations of copper in stream environments, following aerial application of copper to control *Dothistroma* using current management practices, spray technologies, and lower application rates. We hypothesize that under these conditions, the highest risk of copper detection in the stream environments will be either on the day of application or during any rainfall events shortly thereafter.

## Methods

### Trial sites

**Fig. 1:**
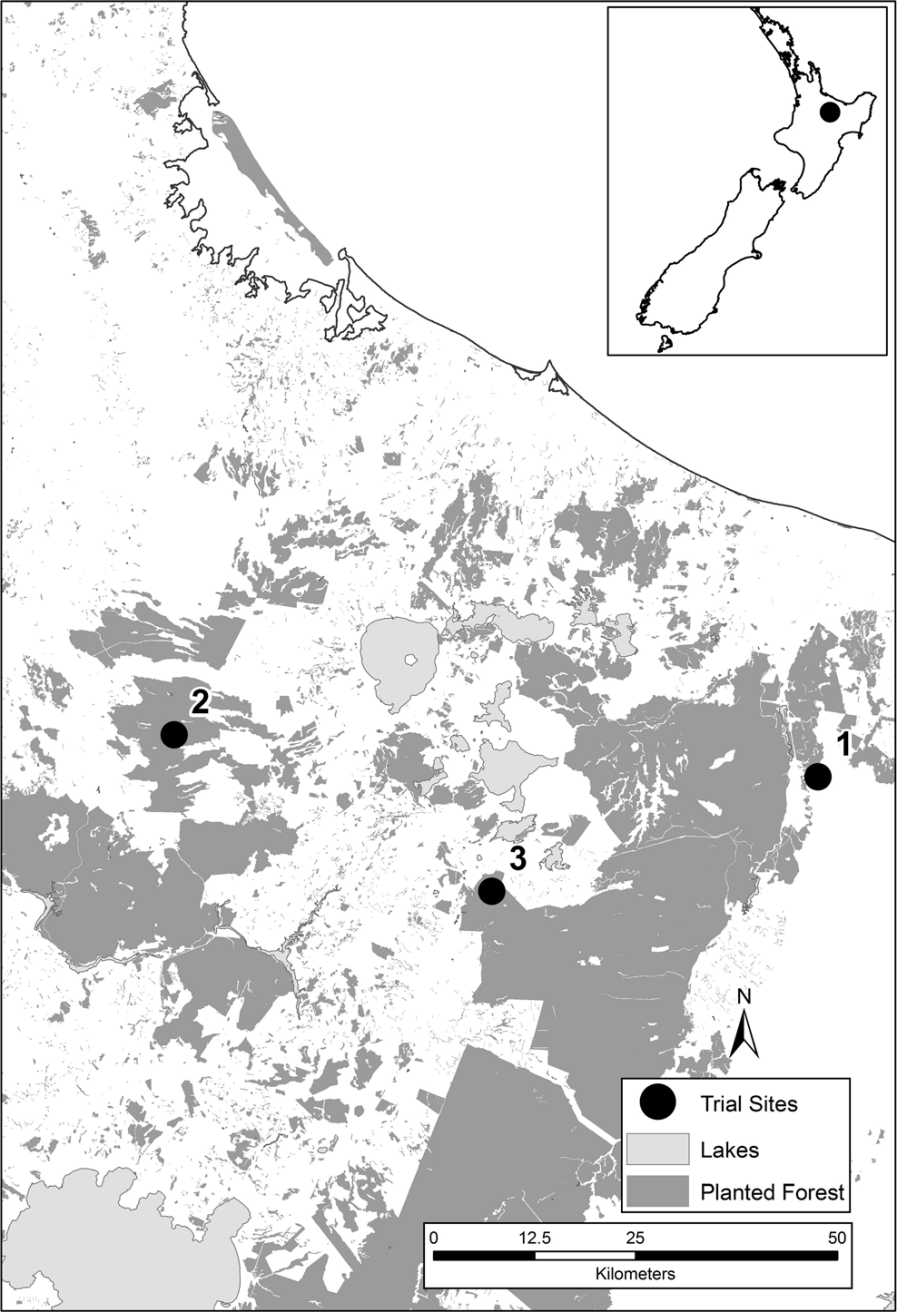
Location of the three trial sites in the central North Island region of New Zealand

The three trial sites were located in the central North Island region of New Zealand, a region highly susceptible to dothistroma needle blight (Bulman et al. 2004) (Fig. 1). The sites were selected to cover the age classes when the trees are most susceptible to *Dothistroma* infection (Bulman et al. 2004) and ranged from 4 to 15 years in age (Table 1). While the upstream catchments at sites 2 and 3 contained a range of age classes (Table 1), the main age class in the immediate trial area was 8–9 years at site 2 and 15 years at site 3. All three sites were underlain by volcanic geology and soils and the topography ranged from strongly rolling to steep hill country (Table 1).

**Table 1:** Characteristics of the three trial sites

	Site 1	Site 2	Site 3
Geology^a^	Rhyolitic ignimbrite	Rhyolitic ignimbrite	Rhyolitic deposits/rhyolitic ignimbrite
Soils^b^	Orthic Pumice Soils	Orthic Pumice Soils	Orthic Pumice Soils
Topography	Moderately steep hills	Moderately steep to steep hills	Strongly rolling to moderately steep hills, undulating in the headwaters
Forest rotation	2nd	2nd	3rd
Stand age (years)	4	8, 9, and 15	7, 8, and 15
Mean stream wetted width (m) W^upstr^/W^downstr^	1.61/2.24	2.25/2.06	3.68/3.18
Mean stream flow (L s^−1^) W^upstr^/W^downstr^	11.1/31.1	118.6/117.0	263.6/360.5
Catchment area (ha)	213	1307	717
Area treated (ha)	213	67.22	514
% catchment treated	100	5	72

*W* ^*upstr*^ upstream monitoring point, *W* ^*downstr*^ downstream monitoring point

^a^ Leonard et al. 2010

^b^ Landcare 2016

The trial sites were in their second or third forest rotation (Table 1), and due to a number of forest ownership changes, historical data on previous copper applications at these sites was limited. At site 1, there was no prior information on copper application during the first forest rotation and the first copper application in the second rotation was applied during this trial. Similar to site 1, no historical information was available on copper treatments in the first rotation for site 2. Available records from 2008 onward showed that parts of the trial catchment had received prior applications of copper annually from 2008 to 2015, excluding 2009. The catchment at site 3 has a history of dothistroma and treatment applications in infected stands within the catchment were estimated at four per rotation.

Catchment areas upstream of the water quality monitoring points at the three trial sites ranged from 213 ha to over 1300 ha, with the site 1 catchment having the smallest streams and lowest flows (Table 1). At site 3, the stream provided a drinking water supply to the nearby Wai-o-tapu Camp. As the water quality characteristics at the upstream (W^upstr^) and downstream (W^downstr^) sampling points were similar at all three sites, only the water quality data from W^upstr^ has been shown in Table 2. Water quality was relatively stable at the time of sampling over the trial monitoring period (see “Data collection” section for details), as indicated by the small standard errors for most variables. The pH was similar between the three sites and within the typical range for New Zealand’s running waters (Davies-Colley and Wilcock 2004). The alkalinity figures indicate that the buffering capacity was low with sites 1 and 3, similar to the median for New Zealand rivers (Maasdam and Smith 1994; ANZECC 2000). The hardness figures were indicative of soft waters in all three streams (ANZECC 2000), also typical of New Zealand’s streams and rivers, although at the low end of the range for site 2 (Hickey 2000). Total suspended solids (TSS) were higher in site 2 compared with the two other sites and dissolved organic carbon (DOC) was comparatively lower at site 3 (Table 2) and below detection limits for most of the trial duration.

**Table 2:** Mean water quality characteristics of the three trial sites at the upstream monitoring point (W^upstr^) (± standard error) (*n* = 6)

	pH	Alkalinity as CaCO_3_	Hardness as CaCO_3_	TSS	DOC
		g m^−3^	g m^−3^	g m^−3^	g m^−3^
Site 1	7.6 (± 0.0)	26 (± 0.0)	18 (± 0.0)	1.5 (± 0.0)	1.8 (± 0.7)
Site 2	7.2 (± 0.1)	15 (± 0.4)	6 (± 0.0)	16.2 (± 2.6)	2.0 (± 0.3)
Site 3	7.1 (± 0.1)	26 (± 0.2)	26 (± 0.1)	1.8 (± 0.3)	0.4 (± 0.2)

*TSS* total suspended solids, *DOC* dissolved organic carbon, *CaCO*_*3*_ calcium carbonate

### Dothistroma treatment

**Fig. 2:**
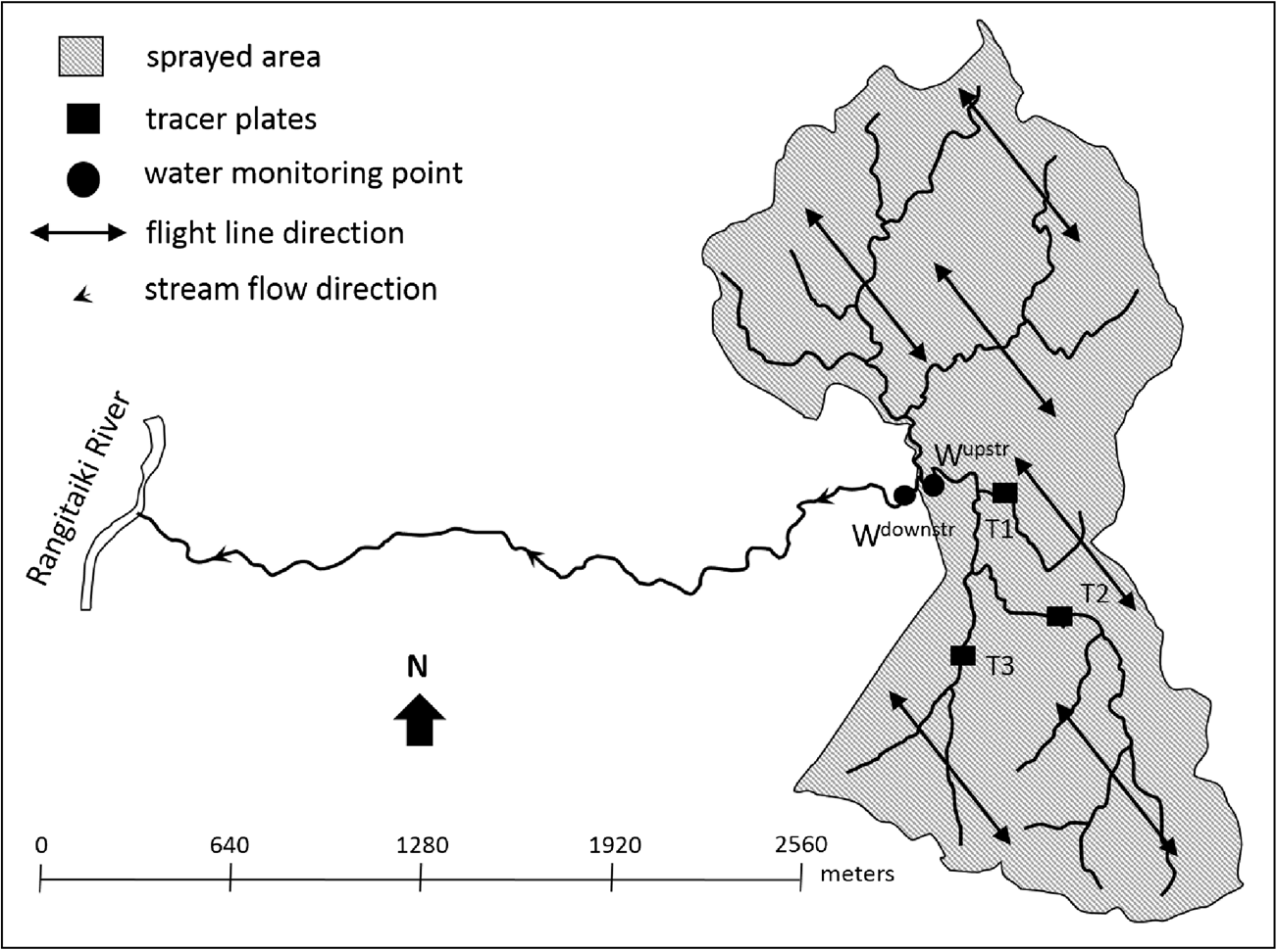
Site 1 showing the area treated with copper spray, the location of the water quality monitoring points, and the position of the tracer plates

**Fig. 3:**
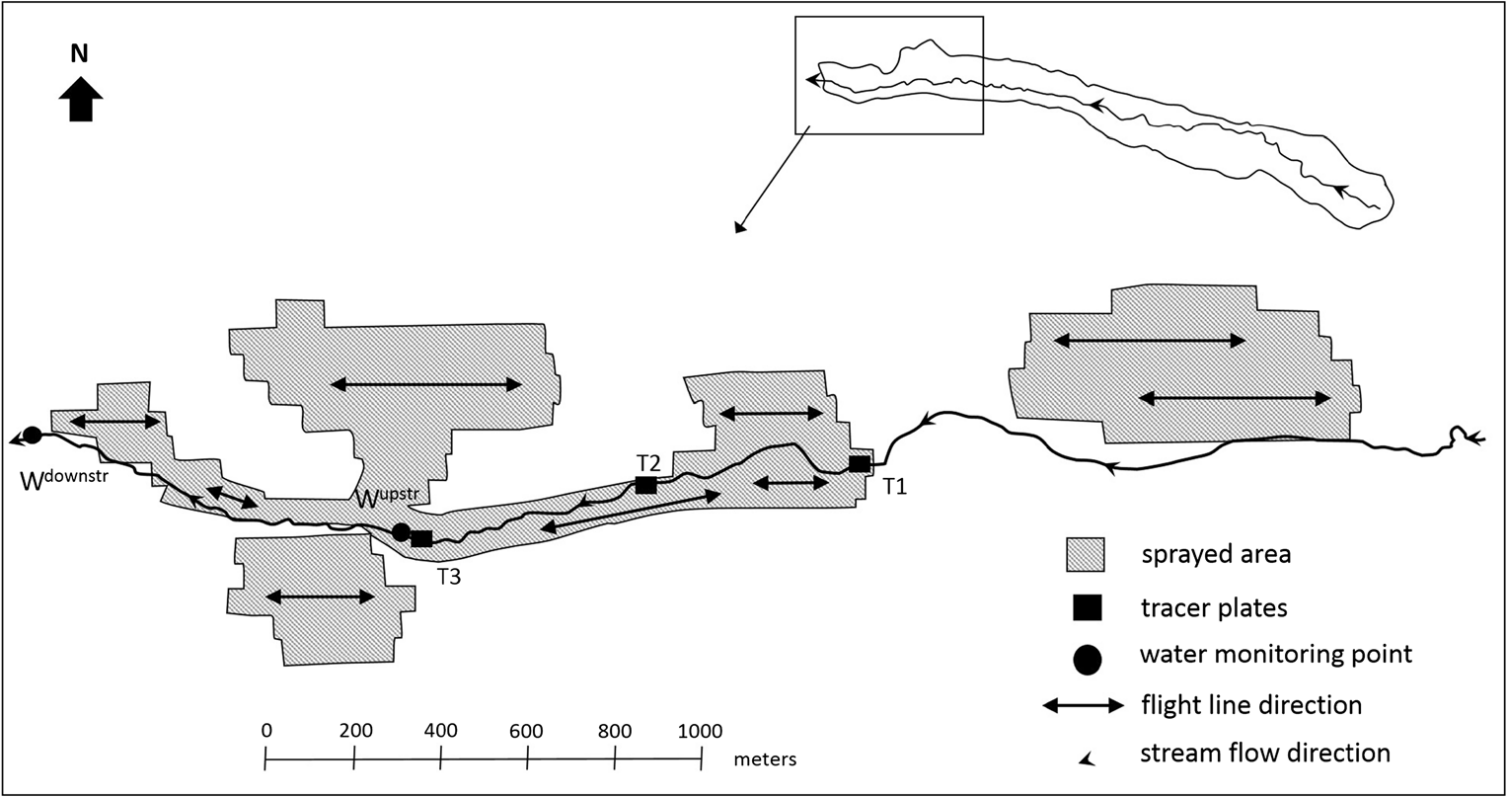
Site 2 showing the area treated with copper spray, the location of the water quality monitoring points, and the position of the tracer plates

**Fig. 4:**
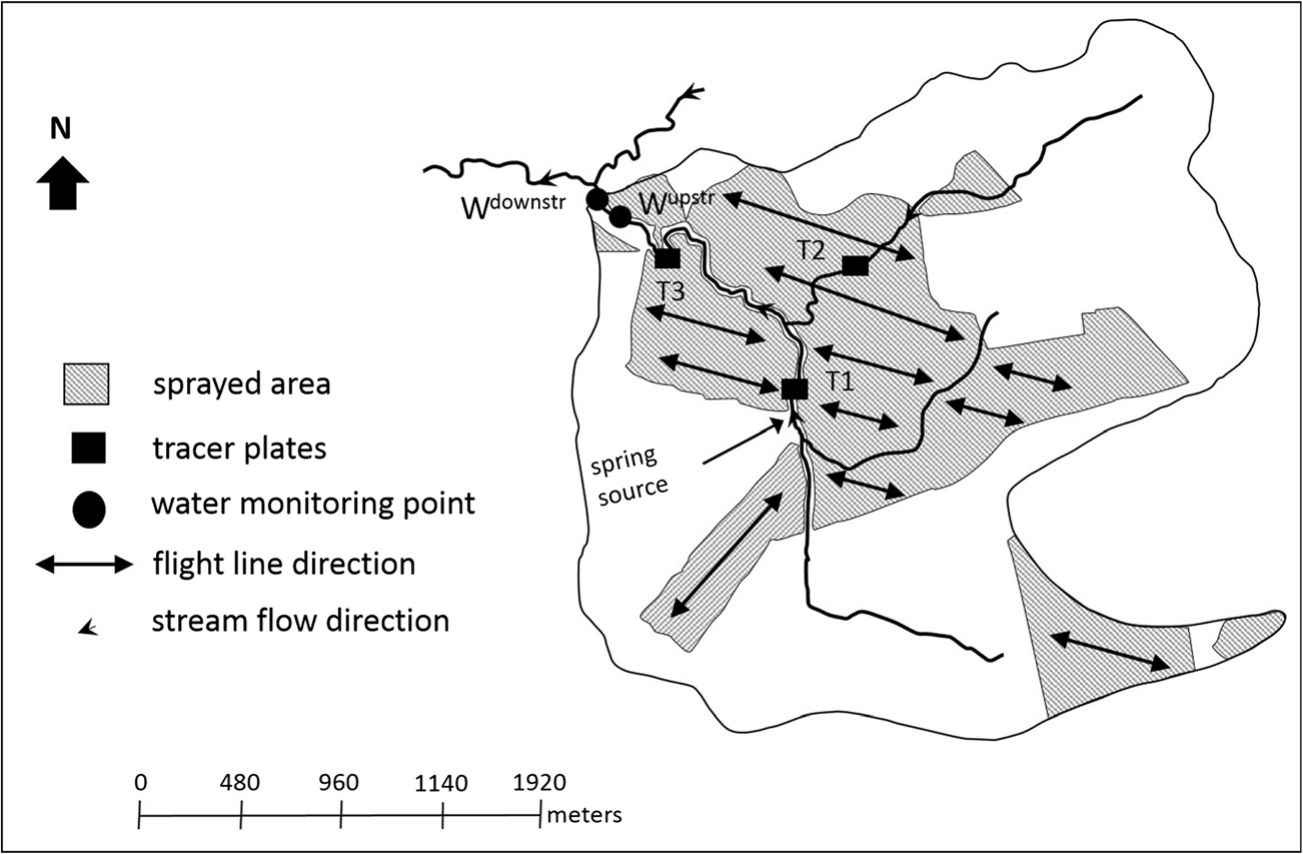
Site 3 showing the area treated with copper spray, the location of the water quality monitoring points, and the position of the tracer plates

All three sites were sprayed by helicopter (Table 3) in mid-November 2015 with either 1.14 kg 75% active ingredient (ai) cuprous oxide (sites 1 and 3) or 1.125 kg 75% ai cuprous oxide (Ag Copp® 75) (site 2) in 2 L of mineral spray oil with additional water to make up to a 5 L ha^−1^ solution (copper spray) (Table 3). Spray oil was added to copper mix to reduce the evaporation of the fine spray droplets and improve the effectiveness of the copper treatment (Bulman et al. 2004). A small droplet size (Table 3) was used to promote the penetration and coverage of the copper spray into the stands. The areas treated with copper upstream of the water monitoring points ranged from 67 to 514 ha with the percentage of the upstream catchment area treated ranging from 5 to 100% (Table 1). At site 1, the flight line direction was across the stream channel within the treated area (Fig. 2). At site 2, flight lines ran parallel to the stream channel and included the stream channel in the upstream section of the trial area (Fig. 3). The sprayed area immediately upstream of the trial site, on the true right of the stream channel (Fig. 3), was treated on the same day as the trial site and flight lines were parallel to the stream channel (Fig. 3). An additional 1 ha was treated on the northern boundary of the catchment (not shown on the map). At site 3, a “no spray” zone was maintained along both sides of the main stream channel (Fig. 4) and averaged approximately 35 m along the perennial stream edge.

**Table 3:** Copper spray aerial application details for three trial sites

Helicopter	Iroquois Bell UH1H
Nozzles	Micronair
Target droplet size	60 μm
Boom length	14.6 m
Swath width; overlap	60 m; 1/2 overlap
Average ground speed	65 knots
Spray release height	12–15 m

### Data collection

#### Meteorological data

A Campbell Scientific Inc. data logger (model CR1000) meteorological station was established at each of the three sites prior to treatment to record five meteorological parameters: air temperature (°C), relative humidity (%), wind direction (degrees) and speed (m/s) at approximately 2.5 m height above ground, and rainfall (mm), logged at 15-min intervals, and monitored for the duration of the trial (1 month after copper application). The data was collated into three output tables of 15 min, hourly, and daily averages and totals, and used to measure the weather conditions at each site on the day of copper spray application and the rainfall events in the following month after treatment.

#### Copper tracer plates

Tracer plates (Mylar® sheets, 105 mm × 148.5 mm) were used to estimate the quantity of copper spray reaching the water surface. At each site, prior to copper application, three sets of 10 tracer plates were spaced horizontally along the stream channel, above the water surface within the treated area (Figs. 2–4), approximately 2 m apart. The majority of tracer plates were positioned at an estimated height above the water surface of 0.1–0.3 m up to a maximum estimated height of 0.8 m. After copper application, the tracer plates were recovered from the stream channel, and each plate was put into a zip-lock plastic bag and transported to the laboratory. A general description was taken on the composition and height of the riparian vegetation along the 20-m sections of the stream channel where the tracer plates were deployed.

### Water and sediment sampling

Two stream monitoring points were established at each of the three sites (Figs. 2–4). The upstream monitoring point (W^upstr^), located at the downstream end of the main area treated with the copper spray, was the main point for water and sediment sampling. The second monitoring point (W^downstr^) was located at varying distances downstream from W^upstr^ (≈ 50 m, 890 m, and 110 m; sites 1–3, respectively) to assess downstream dilution and was sampled at a lower frequency for water only. For all three sites, water samples (250 mL) were taken for copper analysis at W^upstr^ and W^downstr^ twice before the sites were treated. On the day of copper spray application, at each of the three sites, stream water was sampled for copper at W^upstr^ at 15-min intervals in the first hour after application and at half-hourly intervals for a further 3 h. After the day of application, further water samples were taken for copper analysis 1 day after treatment (DAT), and again at 2, 3, 4, 14, or 15 and 30 DAT. At W^downstr^, a single water sample for copper analysis was taken on the day of copper spray application, 2 h and 18 min, after water monitoring started at W^upstr^ at site 1 and within an hour of water monitoring starting at W^upstr^ at sites 2 and 3. Further water samples for copper analysis were taken at W^downstr^, 15 and 30 DAT. Additional water samples were taken at W^upstr^ and W^downstr^ during the first rainfall event after copper spray application, equating to 7, 2, and 6 DAT for sites 1–3, respectively. Water samples (1 L and 125 mL) for water quality analysis were collected from all three sites at W^upstr^ and W^downstr^, twice prior to copper spray application, on the day of application, at 15 and 30 DAT, and on the day of the rainfall event. At all three sites, flow was measured at W^upstr^ and W^downstr^ on all sampling occasions using a Hach FH950 portable velocity meter.

Stream sediment samples were taken at sites 1 and 2 only, as access and water depth precluded sampling at site 3. Sediment samples were taken at W^upstr^, twice prior to copper spray application, on the day of application and at 15 and 30 DAT. All water and sediment samples were labeled, chilled, and couriered in insulated containers to the laboratory accompanied by a chain-of-custody form.

### Laboratory analyses

Initial method development experiments were undertaken by Veritec Laboratories (Scion, Rotorua, New Zealand; http://www.scionresearch.com/general/facilities-and-collections/veritec-laboratories) to determine the amount of copper on the Mylar® sheets, using a wide range of calibration standards. The detection limit of 0.25 ppm was determined. Copper standards were run on the AAS (atomic absorption spectroscopy) (Varian, SpectrAA 220 FS) using test samples along with running repeat AA runs on a number of samples to determine the repeatability of results. The amount of copper recovered from 10 test plates did not differ significantly from the amount applied using a *t* test, indicating that the adsorption capacity of the Mylar® sheets was negligible. Copper spray deposits on the Mylar® sheets were extracted in 50 mL of extract solution (1:1 10% hydrochloric acid/methanol) in double sealed bags, using a reciprocal shaker at 100 rpm for 24 h. Approximately 10 bags were placed into a container (lying flat with sealed end facing up). Each sample extract was decanted into a labeled 50 mL falcon tube. Extract solutions were analyzed for Cu by AAS at 327.4 nm, using standards in range—0.25, 0.5, 1, 2, 5, 10, 15, 20, and 30 ppm.

The surface copper content was calculated in milligrams per liter using the following calculation:

$$ Cu\  concentration from\  AA\ (ppm)\times \left(\ast volume of wash solution in\  bag\ (L)\right). $$

The moisture content was reported separately and not included in the above calculation.

Results less than 0.01% *w*/*w* indicate no copper on surface of sample.

*Samples were double bagged to prevent leakage during extraction. If there was a hole in a bag, the volume of eluent remaining in the bag was measured. The volume of wash solution was adjusted in the calculation for surface copper content if the sample bag leaked.

The water and sediment samples were analyzed by RJ Hills Laboratory (RJ Hill Laboratories, Hamilton, New Zealand; http://www.hill-laboratories.com/) using a Nexion 300D. Water samples were analyzed for total copper, total calcium, and total magnesium (APHA 3125B nitric acid digestion, inductively coupled plasma–mass spectrometry (ICP-MS), screen level, detection limits in water (DL) = 0.011, 1.1, and 0.42 g m^−3^, respectively), total alkalinity (APHA 2320 B, modified for alk <20, DL = 1.0 g m^−3^ as CaCO_3_), total hardness (APHA 2340 B, DL = 1.0 g m^−3^ as CaCO_3_), pH (APHA 4500-H + B, DL = 0.1 pH units), TSS (APHA 2540 D, DL = 3 g m^−3^), and dissolved organic carbon (DOC) (APHA 5310C (modified), APHA 2012, DL = 0.5 g m^−3^). Sediment samples were prepared by air drying at 35 °C, then sieved to obtain the < 2-mm fraction and analyzed for copper using the method of US EPA 200.2 (nitric/hydrochloric acid digestion, ICP-MS, trace level) (Martin et al. 1994). The detection limit for copper in sediment was 0.2 mg kg^−1^ (parts per million). Hills Laboratory is IANZ (International Accreditation New Zealand) Accredited to ISO/IEC 17025 which incorporates the aspects of ISO 9000 relevant to testing laboratories.

## Results

### Site 1

**Fig. 5:**
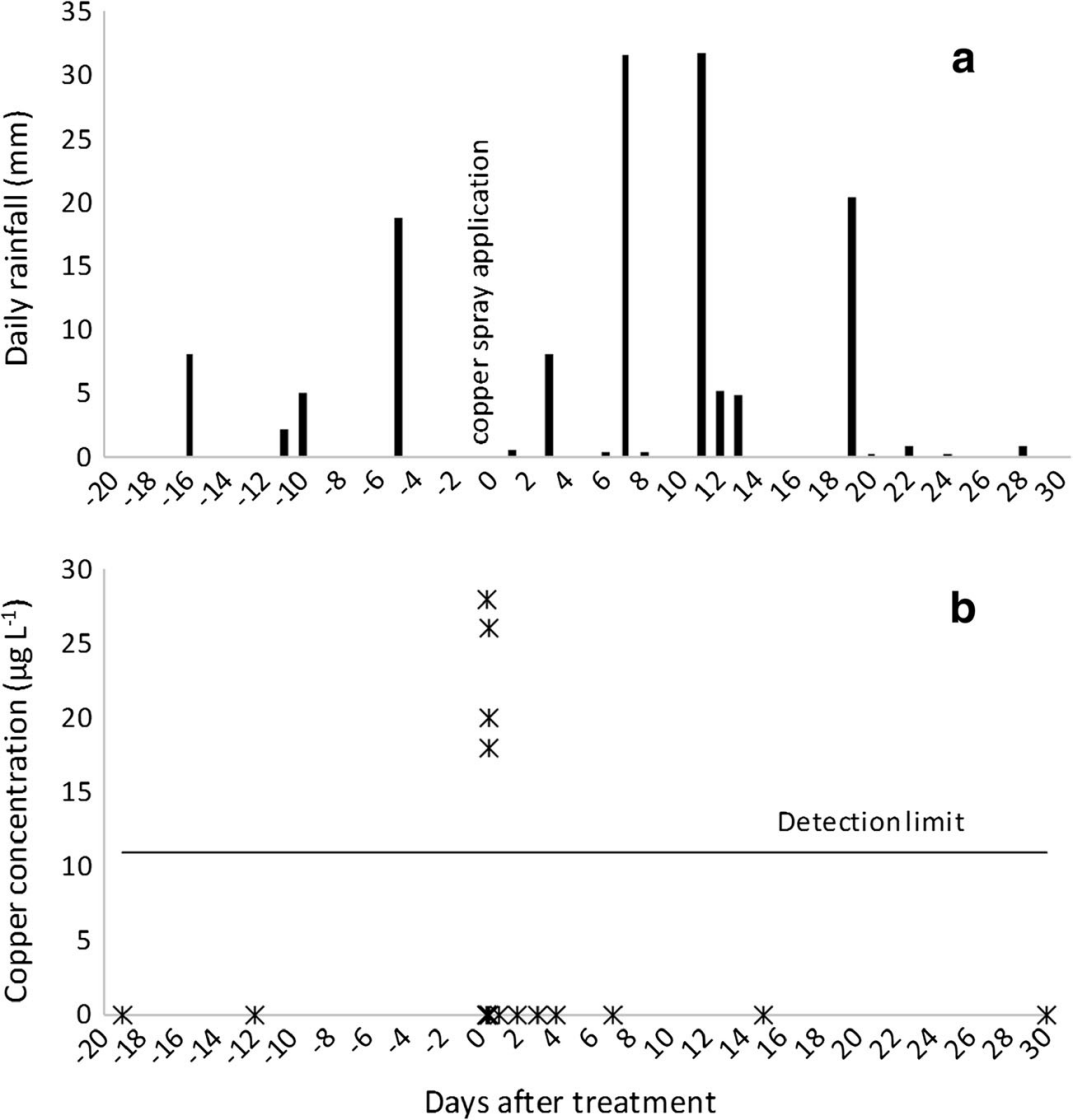
Rainfall (a) and copper concentrations in the stream water (b) at site 1—W^upstr^ during the trial period

On the day of copper spray application, the air temperature averaged 11 °C, relative humidity 83%, wind speed 0.90 m s^−1^, wind direction was variable, and no rainfall was recorded (Fig. 5a). Weather conditions were within the forest company’s prescribed limits for aerial spraying for dothistroma control. The total rainfall for the trial period was 139.2 mm and the highest daily rainfalls of 31.6 and 31.8 mm occurred 7 and 11 days after copper spray application (Fig. 5a).

The three 20-m sections of stream channel where the tracer plates were laid out (Fig. 2) were approximately 0.5–1.0 m in width. Riparian vegetation height varied from around 1.1 to 4 m and overhung the stream channels for most of their length. Key species present included pampas (*Cortaderia* spp.), gorse (*Ulex europaeus*), pate (*Schefflera digitata*), and *Coprosma* species. Transect 3 also had a layer of logging slash covering much of the stream channel. These streams recorded some of the lowest average concentrations of copper collected on the tracer plates (Table 4).

**Table 4:** The average and range of concentrations of copper deposits (kg ha^−1^) collected from the three sets of tracer plates (T1–T3) at the three trial sites (sites 1–3)

	Site 1	Site 2	Site 3
T1	0.5 (0.1–1.8)	0.62 (0.04–1.1)	0.7(0.04–1.5)
T2	0.3 (0.1–0.5)	2.13 (0.9–4.2)	0.3 (0.02–1.1)
T3	0.2 (0.03–0.5)	2.48 (0.6–6.9)	0.1 (<DL^a^–0.2)

The sample size for all sets of tracer plates was 10, except for site 3/T3 where *n* = 9

^a^<DL = below detection limit of 0.25 ppm

Copper concentrations were below the analytical detection limit in the stream water at site 1 at W^upstr^ prior to copper spray application (Fig. 5b). Copper concentrations at W^upstr^ peaked on the day of application at 28 μg L^−1^ and were detected for a period of 1.5 h. Copper concentrations remained below detection limits for the remainder of the trial period (1 month) including the first rainfall event sampled after copper application (29 mm in the preceding 24 h; 7 DAT). The downstream site (W^downstr^) encompassed the larger catchment area that was treated with copper spray (Fig. 2). However, copper concentrations were below the analytical detection limit in stream water at W^downstr^, located approximately 50 m downstream from W^upstr^, even though the sample was taken when copper was still being detected at W^upstr^. Copper concentrations remained below detection limits in stream water at W^downstr^ for the remainder of the trial period.

### Site 2

**Fig. 6:**
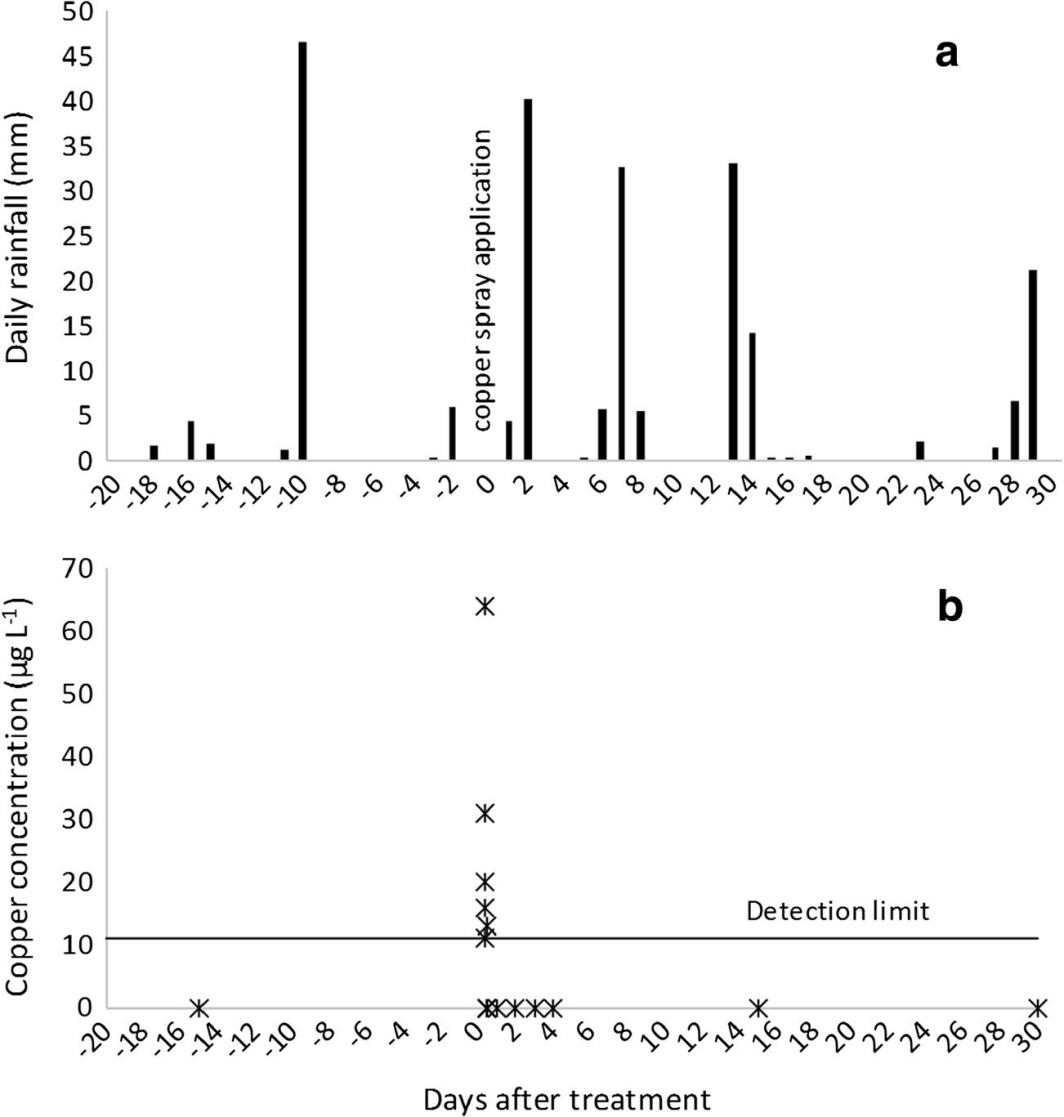
Rainfall (a) and copper concentrations in the stream water (b) at site 2—W^upstr^ during the trial period

On the day of copper spray application, the air temperature averaged 9 °C, relative humidity 67%, wind speed 0.52 m s^−1^, wind direction tended W–NW, and no rainfall was recorded (Fig. 6a). Weather conditions were within the prescribed weather parameters for aerial spraying. The total rainfall for the trial period was 248.4 mm, the highest for all three sites. Although the highest daily rainfall of 46.6 mm occurred 10 days prior to copper spray application (Fig. 6a), the highest post-treatment rainfall of 40.4 mm occurred 2 days after the copper application (Fig. 6a).

The mean stream widths along the 20-m sections of stream channel where the three sets of tracer plates were deployed ranged from ≈ 1.5 to 2.0 m. Riparian vegetation height was variable, ranging from 1.2 to 2.8 m. Vegetation overhanging the channel was interspersed with small patches of more open channel with low vegetation. The main species present were pampas, Himalayan honeysuckle (*Leycesteria formosa*), and blackberry (*Rubus fruticosus*). Some of the highest average copper concentrations were recorded on the tracer plates at this site (Table 4) particularly at T2 and T3.

Copper concentrations were below the analytical detection limit in the stream water at site 2 at W^upstr^ prior to copper spray application (Fig. 6b). Copper concentrations at W^upstr^ peaked on the day of copper application at 64 μg L^−1^ (Fig. 6b), about twice that of site 1, and were also detected for a period of 1.5 h. Copper concentrations remained below detection limits for the remainder of the trial period (1 month) including the first rainfall event sampled after copper application (34 mm in the preceding 24 h; 2 DAT). Copper was detected at W^downstr^ (890 m downstream of W^upstr^) on the day of copper spray application only (12 μg L^−1^). Copper spray was applied along the true right of the stream channel at, and upstream of, W^downstr^ (Fig. 3) which most likely contributed to this result.

### Site 3

**Fig. 7:**
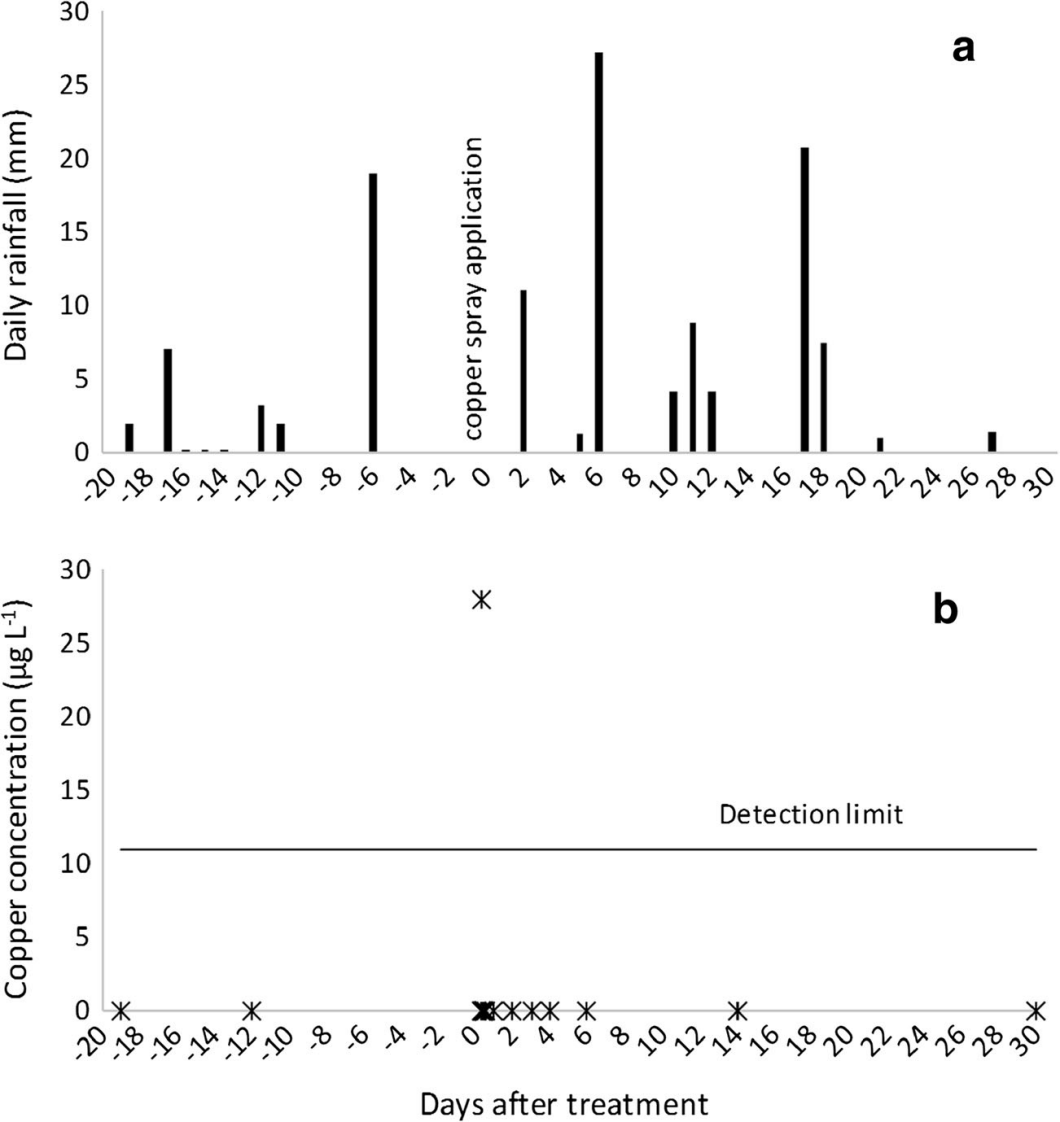
Rainfall (a) and copper concentrations in the stream water (b) at site 3—W^upstr^ during the trial period

On the day of copper spray application, the air temperature averaged 16 °C, relative humidity 79%, wind speed 0.1 m s^−1^, wind direction was predominantly NE–NW, and no rainfall was recorded (Fig. 7a). Weather conditions were within the forest company’s prescription requirements. The total rainfall for the trial period was 121 mm, and the highest daily rainfall of 27.7 mm occurred 6 days after copper spray application (Fig. 7a).

For the first two sets of tracer plates (T1 and T2, Fig. 4), the stream widths along the 20-m sections of channel were approximately 1.0–1.5 m. The stream was wider at T3 (Fig. 4) at approximately 3.0–4.5 m in width. At T1, riparian height varied from 0.7 to 3.8 m along with a few cabbage trees (*Cordyline australis*) estimated at 6 m in height and was comprised mainly of dead and alive reeds and sedges, barberry (*Berberis darwinii*), pongas (*Cyathea* or *Dicksonia* sp.), and blackberry. Canopy closure varied and there were sections of open channel. At T2, the riparian vegetation was lower at 0.1–2.0 m in height and blackberry covered most of the stream channel. At T3, riparian vegetation heights ranged from 1.3 to 3.4 m with the exception of a few *P. radiata* and the vegetation was mainly barberry, along with pongas and blackberry.

Mean copper concentrations varied between the sets of tracer plates at this site (Table 4). Both T1 and T3 were located along the section of stream channel where a “no-spray” buffer was retained (Fig. 4).

Copper concentrations were below the analytical detection limit in the stream water at site 3 at W^upstr^ prior to copper spray application (Fig. 7b). Copper concentrations at W^upstr^ were transient and only detected in one sample (28 μg L^−1^) (Fig. 7b), most likely attributable to the “no-spray” zone along the main stream channel and the high flows in this spring-fed stream (Table 1), rapidly transporting and diluting any copper that reached the waterway. In addition, site 3 had a shorter length of perennial stream exposed to the copper spray (originating at the spring source) (Fig. 4) compared with the two other sites. Copper concentrations at W^upstr^ remained below detection limits for the remainder of the trial period (1 month) including the first rainfall event sampled after copper application (27 mm in the preceding 24 h; 6 DAT). Copper concentrations were below the analytical detection limit at W^downstr^ on the day of copper application, located approximately 110 m downstream from W^upstr^. W^downstr^ was sampled 7 min prior to the one sample at W^upstr^ that recorded the presence of copper and may have missed a possible transient spike of copper down the stream system. Copper concentrations remained below detection limits in stream water at W^downstr^ for the remainder of the trial period.

### Copper concentrations in sediment—sites 1 and 2 only

Copper was detected in the sediment samples at sites 1 and 2 prior to copper application (Table 5). Copper concentrations increased on the day of fungicide application at both sites and remained higher than pre-spray concentrations 1 month after copper application (Table 5). However, the small sample size precluded the ability to test for statistical significance.

**Table 5:** Concentrations of copper in sediment at sites 1 and 2, prior to and after copper application

	Pre-spray 1	Pre-spray 2	Spray day	Post-spray 15 DAT	Post-spray 30 DAT
Site 1 (mg kg^−1^ dry weight)	1.7	1.8	2.6	4.4	3.6
Site 2 (mg kg^−1^ dry weight)	2.8	4.1	6.1	5.0	5.0

No sediment samples were taken at site 3

*DAT* days after treatment

## Discussion

### Copper concentrations in stream water

Regardless of the tree age, stream size, stream flow, riparian composition, flight line direction, or leaving a “no spray” buffer along the stream edge, copper was detected in the stream water for a short duration at all three sites (up to 1.5 h) on the day of copper application. Given that a “spray drift” method is used to facilitate the penetration of copper into the infected stands, particularly along stand edges (Bulman et al. 2004), the potential exists for some copper to reach the waterway, even when a “no-spray” buffer is retained. The amount of copper that was detected on the tracer plates within the treated areas provided a measure of drift into the stream channel and appeared to be most strongly influenced by the density of the riparian vegetation and the extent of riparian canopy closure, in combination with the stream size. For example, some of the lowest concentrations of copper on the tracer plates occurred at sites 1 and 3 in two small streams that had extensive riparian cover and, at site 1, additional cover from logging slash, even though the flight direction was across the stream channel (Table 4). In contrast, even though a “no-spray” buffer was retained along the stream channel at T1, the higher concentrations on some tracer plates reflected the areas of open water along this section of stream channel.

Copper concentrations were below the analytical detection limit at the three sites during the first rainfall event after copper spray application which occurred anywhere from 2 to 7 days after treatment. There is the potential for copper wash-off into waterways during rainfall events that occur before the copper spray has dried. The quick drying and adhesive properties of the copper spray solution should minimize this window of risk, and given sufficient time to dry, copper wash-off from vegetation should be minimal (Bulman et al. 2004). Copper weathering over time provides another potential source of copper residues. Field trials indicate that most of the copper is weathered from the pine needles within 3 months of application (Gilmour and Noorderhaven 1973). Any copper residues reaching the ground bind strongly to organic matter in soil, minimizing the risk of leaching into waterways (Kiaune and Singhasemanon 2011). However, this does not preclude the risk of copper transfer to waterways during high rainfall events that initiate run-off and erosion.

The short duration of copper detected in the water column on the day of copper spray application, and the minimal concentrations detected at the downstream sampling points indicated rapid dilution, absorption, and adsorption of copper within the stream systems. While some copper adsorbs to particulate matter, copper has a strong affinity to the dissolved constituents in the water which are usually present in higher quantities than copper in freshwater systems, hence the majority of the dissolved copper is removed from the water column via this process (Kiaune and Singhasemanon 2011). The amount of copper remaining in a biologically available form, primarily the cupric ion Cu^2+^, will be influenced by the complex interactions of a wide range of physical and chemical factors. In general, the amount of bioavailable copper and toxicity risk to aquatic organisms declines with increasing water hardness, alkalinity, and DOC, with DOC an important influence on copper availability. While some studies show that dissolved copper declines with increasing pH, there is conflicting information on the effect of pH on copper toxicity (ANZECC 2000; Environmental Protection Agency 2007; Kiaune and Singhasemanon 2011).

The water quality samples taken over a 6-week period provided an indication of the water quality characteristics at the three trial sites (Table 2). Based on this data, there was the potential for most of the copper in these streams to be complexed by DOC, which is usually present in sufficient concentrations in most water bodies, to remove the biologically available copper to below toxic levels. However, site 3 had very low DOC levels and could be more susceptible to copper inputs. In addition, the soft waters and the low alkalinity in these streams increase the likelihood of copper being present in biologically available and more toxic forms for uptake by aquatic organisms (ANZECC 2000; Environmental Protection Agency 2007; Kiaune and Singhasemanon 2011).

The peak copper concentrations measured at the three sites during this study (range 28–60 μg L^−1^) following aerial application were an order of magnitude lower than those measured in two unpublished New Zealand studies in the 1970s and 1980s (peak concentrations 210 and 300 μg L^−1^) (Collier and Hickey 1998; unpublished data, P. Beets, Scion, Rotorua, New Zealand) and concentrations predicted from a desktop exercise under an average and worst-case scenarios (see Collier and Hickey 1998 for scenario details). Since this research was undertaken, there has been considerable advancement in spray application technologies, aerial guidance systems, copper formulation performance, and application rates (Bulman et al. 2016), reducing the risk of copper input into waterways.

The copper concentrations from this study were also low compared with copper concentrations in global surface waters, which ranged across several orders of magnitude and were influenced by variations in the water quality characteristics and, more importantly, anthropogenic inputs. In Bowen’s (1985) review, copper concentrations in freshwater ranged from < 0.2 to 135 μg L^−1^ (median 3 μg L^−1^). Dorsey et al. (2004) reported copper concentrations in rivers ranging from 0.5 to 1000 μg L^−1^ and copper concentrations across a range of drinking water sources in Canada ranged from ≤ 5 to 530 μg L^−1^ (Meranger et al. 1979). While natural or background copper concentrations in surface waters were at the lower end of this range (0.11–10 μg L^−1^) (Förstner and Wittmann 1983; ANZECC 2000; Dorsey et al. 2004), concentrations can increase by several orders of magnitude when influenced by anthropogenic inputs such as municipal and industrial waste waters, piggery effluent, and mining (Smith and Williamson 1986; Dorsey et al. 2004). Some of the highest copper concentrations have originated from the mining industry (≈ 200,000 μg L^−1^) (Environmental Protection Agency 2007). In comparison, the concentrations of copper measured in this trial were at the lower end of the range associated with anthropogenic activities.

### Copper in sediment

Copper persisted for longer in stream sediment at the two sites where it was monitored. Copper forms relatively strong bonds in sediment, primarily bound to the organic content of the pore water (Kiaune and Singhasemanon 2011). Similar to water, copper speciation processes in sediment are complex and influenced by a range of factors such as temperature, oxygen, organic carbon, and sediment characteristics. There is the potential for copper in sediment to be partitioned back into the water column as the organic component decomposes. As the amount of organic matter in sediment pore water tends to exceed the amount of copper, most of the copper is held in organic complexes and the amount of copper recycling back into the water column is likely to be low. Nevertheless, the persistence of copper in sediment increases the exposure time and potential risk for aquatic organisms that reside in benthic sediment environments (Canadian Council of Ministers of the Environment 1999; Kiaune and Singhasemanon 2011).

Sediment is an important sink and storage site for copper (Kiaune and Singhasemanon 2011). Copper sources in sediment are primarily derived from soil transported via run-off and erosion processes (Dorsey et al. 2004). In Canada, copper concentrations in freshwater sediments ranged from 2 to 10,000 mg kg^−1^ (Canadian Council of Ministers of the Environment 1999). Copper concentrations in streambed sediments across the USA ranged from 6 to 620 mg kg^−1^ (median 27 mg kg^−1^). Highest copper sediment concentrations were usually associated with mining, industrial activities, and urban areas (Förstner and Wittmann 1983; Canadian Council of Ministers of the Environment 1999), and in the case of Rice (1999), concentrations in urban stream sediments were higher than in agricultural and forestry areas exceeding Canadian sediment quality guidelines at times.

The copper concentrations measured in stream sediments in this study (range 1.7–6.1 mg kg^−1^; Table 5) were low in comparison to both the background concentrations and concentrations influenced by anthropogenic activities reported on in the international literature. These results are comparable with the concentrations of copper measured in New Zealand planted forest soils in both sprayed and unsprayed stands (mean 4.27 and 2.67 mg kg^−1^, respectively) (Rolando et al. 2016) and also reflect the infrequent use of copper over a 28-year forest rotation (≈ 2–5 treatments; maximum—two treatments in 1 year) (Bulman et al. 2008).

### Risk to human health and the aquatic environment

Copper concentrations in the stream water in this trial were well below drinking water standards (Table 6) and unlikely to pose a risk to human health. Toxicology testing indicates that some of the highest environmental risks are associated with copper in the aquatic environment (Kiaune and Singhasemanon 2011; MacBean 2012), hence the inclusion of cuprous oxide (the copper formulation used in the treatment of dothistroma in New Zealand) in the FSC highly hazardous pesticide list based on its toxicity to aquatic organisms (Table 6) (Forest Stewardship Council 2015a, 2015b). FSC have referenced the toxicity threshold in MacBean (2012) to classify cuprous oxide as highly hazardous (LC_50_ 18.9 μg L^−1^ over 48 h using *Daphnia* as the test organism). In these field studies, copper concentrations did exceed 18.9 μg L^−1^ for approximately 1.5 h or less, across all three sites, well below the 48 h acute toxicity time frame. The copper EC_50_ values for several New Zealand aquatic invertebrate species (Hickey 2000) also indicated a low risk to aquatic organisms based on the short exposure times to the concentrations encountered in this study.

**Table 6:** Environmental and drinking water guidelines and regulations for copper in freshwater environments

Drinking water	mg L^−1^ (μg L^−1^)	Country	Reference
Maximum acceptable value^a^	2 (2000)	New Zealand	Ministry of Health 2008
Guideline value^b^	2 (2000)	World	World Health Organisation 2011
Parameter	2 (2000)	Europe	The Council of the European Union 1998
Maximum contaminant level goal (MCLG)^c^	1.3 (1300)	USA	Environmental Protection Agency 2009
Freshwater ecosystems	μg L^−1^		
Trigger value of copper to protect 95% of species^d^	1.4	New Zealand and Australia	ANZECC 2000
Final acute value (dissolved copper)^e^	4.67	USA	EPA 2007
Final Chronic Value (dissolved copper)^e^	1.45	USA	EPA 2007
Copper parameter^f^	2–4	Canada	Canadian Council 2007
Acute toxicity^g^	LC_50_/EC_50_ < 50	International	FSC 2015b
Sediment	mg kg^−1^dry wt		
Interim sediment quality guideline^h^	Low effects–65 High effects–270	New Zealand and Australia	ANZECC 2000
Interim sediment quality guideline^i^	35.7	Canada	Canadian EQG 1999
Probable effects level^i^	197	Canada	Canadian EQG 1999

^a^The highest concentration of copper in the water that, on the basis of present knowledge, is considered not to cause any significant risk to the health of the consumer who consumes 2 L of that water a day over their lifetime (usually taken as 70 years), based on a body weight of 70 kg

^b^The default assumption for consumption by an adult is 2 L of water per day, whereas the default assumption for body weight is 60 kg

^c^The level of a contaminant in drinking water below which there is no known or expected risk to health

^d^Applies to a typical slightly moderately disturbed system. Calculated using a hardness of 30 mg L^−1^ CaCO_3_. Based on chronic data with test durations of days (7–42 days) for a range of aquatic organisms

^e^Recommended ambient acute and chronic dissolved copper concentrations that, when met, will protect aquatic life. Concentrations are based on a reference set of water quality parameters

^f^2 μg·L^−1^ at 0–120 mg·L^−1^ CaCO_3_ (soft to medium hardness); 3 μg·L^−1^ at 120–180 mg·L^−1^ CaCO_3_ (hard); 4 μg·L^−1^ at > 180 mg·L^−1^ CaCO_3_ (very hard)

^g^The Forest Stewardship Council (FSC) highly hazardous pesticide threshold for acute toxicity to aquatic organisms

^h^<65 minimal effect; 65 to <270 possible effects; ≥270 probable effects

^i^<35.7 mg kg^−1^ rarely; 35.7–197 mg kg^−1^ occasionally; >197 mg kg^−1^ frequently associated with adverse biological effects

Copper concentrations in this study were also above the freshwater ecosystem guidelines in Table 6, but the detection limit of 11 μg L^−1^ precluded determining the duration that these guidelines were exceeded from the field data. Regression analysis of the copper decay curves at sites 1 and 2 (insufficient data at site 3) estimated the time for copper concentrations to decline to 1 μg L^−1^ at 6 and 3 h, respectively. These estimated time frames were lower than those typically used in aquatic acute toxicity tests (48–96 h) or tests for chronic effects such as growth rates and reproduction (days to weeks) (Jarvinen and Ankley 1999; European Food Safety Authority 2013).

However, the sub-lethal impacts of copper across a range of end points (i.e., growth, reproduction) also need to be considered (Jarvinen and Ankley 1999; Kiaune and Singhasemanon 2011). For example, for the long-lived European eels subject to long-term metal exposure in a Spanish lake (mean copper concentrations 1.8–274 μg L^−1^), the amount of copper in liver tissue was significantly related to a decrease in the hepatosomatic index, which provides an indication of the energy status of an animal (Esteve et al. 2012). Fish chemosensory mechanisms are particularly sensitive to copper, where damage has been observed in salmon (*Oncorhynchus* spp.) when exposed to copper concentrations ranging from 2 to 20 μg L^−1^ for several hours (Kiaune and Singhasemanon 2011). In addition, Playle et al. (1992) found that copper begins to accumulate on fathead minnow (*Pimephales promelas*) gills within 20 min of aqueous exposure and that “copper deposition on gills was indeed fast, with maximal deposition occurring by about 1 hour...” The half-saturation time for copper binding to the gills of rainbow trout exposed to a range of concentrations may be on the order of 150 to 200 s (Reid and McDonald 1991). Pulsed exposure to copper of varying frequencies and at concentrations similar to the peaks measured in this study resulted in some significant impacts on survival, growth, or reproduction of fish and invertebrates, although exposure times (7 and 14 days) were much longer than in this study (Diamond et al. 2006). Thus, there is the possibility that short pulsed exposures to copper may lead to sub-lethal effects or latent mortality for some aquatic organisms. Additional research is needed to identify the risk of sub-lethal effects of copper to aquatic organisms subject to the concentrations of copper found in freshwater receiving environments following aerial applications to treat fungal diseases in forests.

Copper concentrations in the sediments of the streams in this study were well below sediment quality guidelines (Table 6) indicating a low risk to aquatic environments. A small proportion of adverse effects were observed in Canadian freshwater sediments once copper concentrations reached 15 mg kg^−1^; however, the majority of cases occurred when concentrations exceeded the interim sediment quality guideline (Table 6) (Canadian Council of Ministers of the Environment 1999). Studies on the effects of copper in sediments on benthic dwelling organisms is limited (Jarvinen and Ankley 1999) and has been identified as an information gap by the European Food Safety Authority (European Food Safety Authority 2013).

## Conclusion

Under present-day technologies and the environmental conditions present at these three sites, the aerial application of copper to control dothistroma posed a low risk to the receiving aquatic environment. This work highlights the importance of bench-marking the concentrations of copper in waterways under operational conditions in forests against the toxicology standards identified via laboratory and experimental procedures when assessing the actual risk of copper to the aquatic environment. On-going research and technological advances to further improve aerial application technologies are likely to further reduce this risk (Bulman et al. 2016). The information from this research will assist in informing the debate around the use of copper in forests and the recent decision by FSC to place copper on the highly hazardous pesticide list.

However, this is an initial study and further testing across a wider range of freshwater environments under differing geological, soil, and forest conditions both in New Zealand and overseas is needed to more fully identify the extent of risk to aquatic environments associated with the aerial application of copper. This is particularly important if predicted climate changes result in an increase in the extent and severity of disease outbreaks, infection levels, and (or) a decrease in timber productivity that compromises economic viability. This may necessitate a change from non-treatment to implementing new treatment strategies or strengthening of existing treatment strategies to manage the disease where aerial application of copper becomes a viable treatment option in areas where it is currently not used. Nevertheless, New Zealand’s knowledge in the use of aerial application of copper to control dothistroma could be modified and applied to other regions of the world.
